# Relationship between self-disclosure to first acquaintances and subjective well-being in people with schizophrenia spectrum disorders living in the community

**DOI:** 10.1371/journal.pone.0223819

**Published:** 2019-10-16

**Authors:** Kazuki Yokoyama, Takafumi Morimoto, Satoe Ichihara-Takeda, Junichi Yoshino, Kiyoji Matsuyama, Nozomu Ikeda

**Affiliations:** 1 Department of Occupational Therapy, School of Health Sciences, Sapporo Medical University, Sapporo, Hokkaido, Japan; 2 Department of Clinical Psychology, Faculty of Health Sciences, Kyorin University, Mitaka, Tokyo, Japan; 3 Department of Nursing, Faculty of Health Sciences, Japan Health Care College, Sapporo, Hokkaido, Japan; Centre Hospitalier Regional Universitaire de Tours, FRANCE

## Abstract

**Objective:**

Focusing on people with schizophrenia spectrum disorders living in the community, the present study aims to examine the characteristics of and gender differences in self-disclosure to first acquaintances, and to clarify the relationship between self-disclosure and subjective well-being.

**Methods:**

Participants (32 men and 30 women with schizophrenia spectrum disorders) were examined using the subjective well-being inventory, an original self-disclosure scale for people with mental illness, as well as the Rosenberg self-esteem scale, the Link devaluation-discrimination scale, and the affiliation scale.

**Results:**

The self-disclosure content domains in descending order were as follows: “living conditions,” “own strengths,” “experiences of distress,” and “mental illness and psychiatric disability.” There were no significant gender differences in self-disclosure in the total and domain scores. Multiple regression analyses by gender revealed that: (1) in men, decreasing feelings of ill-being were significantly predicted by self-disclosure about “living conditions,” self-esteem, and perceived stigma; (2) in women, increasing feelings of well-being were significantly predicted by self-disclosure about “own strengths,” self-esteem, and sensitivity to rejection.

**Conclusions:**

Self-disclosure to first acquaintances was related to subjective well-being in people with schizophrenia spectrum disorders living in the community. This result supports the recovery model and the strengths model. It suggests the importance of interventions targeting self-disclosure to first acquaintances about experiences as human beings, such as “living conditions” and “own strengths,” as it relates to subjective well-being in community-based mental health rehabilitation.

## Introduction

Community mental health care has evolved around the world. In Japan, since the Ministry of Health, Labour and Welfare [1] announced its policy to shift away from hospitalized medical treatment, the number of people with mental illness living in the community has gradually increased [2]. However, there remains a harmful mental health-related stigma in Japan. In particular, schizophrenia is more stigmatized than depression, and the severity of the illness increases the stigmatizing attitude toward it [3]. Consequently, people with mental illness are often reluctant to express themselves and avoid social participation [4]. In the present study, we focus on the self-disclosure of people with mental illness, which is important for their recovery.

Self-disclosure is generally defined as an “act of revealing personal information to others” [5] and is an indicator of stable personality attributes, good psychological adjustment, and mental health [5–7]. Since self-disclosure has a positive relationship with self-esteem [5] and affiliative motives, including affiliative tendency and sensitivity to rejection [8], it can be seen as the basis for enhancing well-being. Previous studies exhibited gender differences, in that men are more willing than women to disclose to strangers and acquaintances, but women are more willing than men to disclose to intimates [9]. Previous studies of self-disclosure in the mental health domain have focused on disability disclosure and coming out with a mental illness. Whether to disclose one’s mental disability at work is an important issue [10]. Deciding to disclose one’s personal experiences with mental illness is not an easy decision. Many people cope with perceived stigma by withholding their illness and disability. They are able to shelter their shame by not letting other people know about their mental illness [11]. However, benefits of disclosing disability have also been reported. To recognize disability disclosure as a benefit can diminish the deleterious effects of perceived stigma on quality of life, thereby encouraging people to move toward achieving their life goal [12].

In previous studies, we chose to focus on the different contents of disclosure (e.g., interests, leisure, daily experiences, strength) by people with mental illness and developed a self-disclosure scale for people with mental illness (hereafter “SDMI”) [13]. This scale was developed based on the social penetration model [14], in which the degree of self-disclosure increases with progress in the level of endorsement from others. Our previous study showed that all content domains of the amounts of self-disclosure to close people are positively correlated with subjective well-being [13]. Considering these findings, our study suggests that disclosing one’s “living conditions” and “own strengths” to close people, in addition to disclosing disability, is important for the well-being of people with mental illness.

While the relevance of self-disclosure to close people became clear in our previous study [13], the relevance of self-disclosure to first acquaintances has not yet been clarified. “First acquaintances” refers to people meeting each other for the first time in social situations and with whom one will continue to have a relationship in the future. People with mental illness who live in the community have opportunities to interact with first acquaintances; however, some such people have trouble disclosing personal experiences, especially in more casual relationships. The social penetration model [14] suggests that the amount and depth of self-disclosure are more likely to increase, along with communication of intimate information, when an interpersonal interaction develops toward an intimate relationship. This model leads to the hypothesis that self-disclosure to first acquaintances is lower than that to close people, which may relate to subjective well-being. Additionally, self-disclosure to first acquaintances is difficult for people with mental illness because of public stigma and self-stigma. Under the influence of perceived stigma, it is possible that self-disclosure to first acquaintances differs, in characteristics and its relationships to subjective well-being, from self-disclosure to close people, depending on the content of the disclosure. In clinical practice, it is important to understand self-disclosure to first acquaintances and to support the individual timing and methods of self-disclosure through psychiatric rehabilitation. In particular, this study contributes by clarifying what contents of self-disclosure are associated with subjective well-being.

Focusing on people with schizophrenia spectrum disorders (SSD) living in the community, this study examines the characteristics of self-disclosure to first acquaintances and the related gender differences and clarifies the relationship between self-disclosure and subjective well-being. The present study aims to generate hypotheses about the relationships between self-disclosure to first acquaintances (along with other variables) and the subjective well-being of people with SSD living in the community. A basic assumption of this study is that some domains of self-disclosure to first acquaintances are correlated with subjective well-being (as are other factors previously found to be correlated with subjective well-being), such as self-esteem, perceived stigma, and affiliation motives.

## Material and methods

### Participants

The participants were selected from three psychiatric day-care centers and four employment support offices in Hokkaido, Japan. All participants were diagnosed with SSD by trained psychiatrists (F20-29; ICD-10) [15]. The inclusion criteria were: (i) aged over 20 years; (ii) living in the community without the use of any advocacy services; and (iii) no history of a head injury, mental retardation, or serious medical disease, such as loss of consciousness. We excluded participants who had difficulties understanding ethical considerations and/or the questionnaire items. To calculate the sample size using G*Power (http://www.gpower.hhu.de), we utilized ten predictors with 0.80 power at the 0.05 alpha level and an estimated effect size based on our previous study [13] in reference to Cohen’s proposition [16]. This process revealed that a sample size of at least 30 was required.

### Procedures

The participants completed the self-administered questionnaire. It consisted of demographic variables, the self-disclosure scale, and questions on subjective well-being, in addition to self-esteem, perceived stigma, and affiliative motives, which are all reported to be related to subjective well-being. Before conducting the survey, researchers explained the contents of the questionnaires using simple words and concrete examples to promote correct understanding. During administration of the questionnaire survey, researchers supported the participants so that they could ask questions and fully understand the questionnaire. This survey was conducted from May to November 2014.

### Measurements

#### Demographic and clinical data

The demographic variables were as follows: age, gender, education level, and residence status. The clinical variables comprised mental illness duration and the utilization of psychiatric services. After obtaining the participants’ consent, we asked the staff of each research facility to confirm whether the participants’ questionnaire responses were reliable, such as their age and diagnosis. The staff confirmed that no mistakes were made.

#### Amounts of self-disclosure

Amounts of self-disclosure were measured using the SDMI [13]. Written in Japanese, this scale asks respondents how much they talk about matters included in 23 items across 4 content domains: “living conditions,” “own strengths,” “mental illness and psychiatric disability,” and “experiences of distress” (see S1 Appendix). The SDMI items were created based on qualitative analysis of semi-structured interviews in which 18 participants answered questions concerning “what kind of aspects of self are disclosed in daily living?” [17]. These items were subjected to exploratory factor analysis, resulting in the identification of 23 items distributed among four domains of disclosure content: living conditions, own strengths, mental illness and psychiatric disability, and experiences of distress [13]. A self-disclosure scale using a similar self-measured questionnaire was used in previous research [18,19]. The validity of self-measured data was shown by comparing it to objective data about self-disclosure obtained through observation [18]. This scale demonstrated sufficient internal consistency with a Cronbach’s alpha of 0.93, while the four content domains reported Cronbach’s alphas ranging between 0.79 and 0.86 and showed sufficient test-retest reliability and criterion validity [13]. Participants rated each item using a 5-point Likert scale (1–5), with higher scores indicating greater amounts of self-disclosure. In this study, we set the target for self-disclosure as first acquaintances with whom the participants would continue to have a relationship in the future. Researchers explained this definition to participants and presented specific examples.

#### Self-esteem

Self-esteem was measured using the Japanese version of the Rosenberg self-esteem scale [20]. This scale contains ten items and measures positive attitude toward self. Participants rated each item using a 5-point Likert scale (1–5), with higher scores indicating greater self-esteem. This scale previously demonstrated internal consistency, with the first factor contributing 43% [20].

#### Perceived stigma

Perceived stigma was measured using the Japanese version of the Link devaluation-discrimination scale [21]. This scale contains 12 items and measures perceptions of community residents’ beliefs towards people with mental illness. Each item is framed as “Most people think &” in order to minimize social desirability bias. Participants rated each item using a 4-point Likert scale (1–4), with higher scores indicating stronger perceived stigma. This scale previously demonstrated internal consistency with a Cronbach’s alpha of 0.85 [21].

#### Affiliation motives

Affiliation motives were measured using the questionnaires on affiliation motives [22]. This scale contains 18 items across two sub-scales of “affiliative tendency” and “sensitivity to rejection.” The former indicates the inclination to form and maintain intimate relationships with people. The latter indicates the extent of fear of rejection by others. Participants rated each item using a 5-point Likert scale (1–5), with higher scores indicating greater affiliation motives. The subscales “affiliative tendency” and “sensitivity to rejection” previously demonstrated internal consistency with respective Cronbach’s alphas of 0.86 and 0.88 [22].

#### Subjective well-being

Subjective well-being was measured using a Japanese version of the Subjective Well-Being Inventory (SUBI) [23]. SUBI is designed to measure the feeling of well-being or ill-being as experienced by an individual or a group of individuals in various day-to-day life concerns [24]. This scale contains 40 items across two subscales: “feelings of well-being” and “feelings of ill-being.” Participants rated each item using a 3-point Likert scale (1–3). The total score for feelings of well-being ranged from 19 to 57, with higher scores indicating greater well-being. The total score for feelings of ill-being ranged from 21 to 63, with higher scores indicating lower ill-being. This scale previously demonstrated high internal consistency with Cronbach’s alphas of 0.86 in men and 0.84 in women [25].

### Statistical analysis

Descriptive statistics, including demographic and clinical data, amounts of self-disclosure, self-esteem, perceived stigma, affiliation motives, and subjective well-being were calculated. Internal consistency was measured using Cronbach’s alpha coefficient. Next, variable distribution normality was verified using the Shapiro-Wilk test. The variables departed from the theoretical normal distribution with a slight significance (P<0.05), and so, non-parametric tests were selected. The Mann-Whitney U test was performed to investigate gender differences in each variable. Subsequently, Spearman’s rank correlation coefficients were calculated to investigate correlations between self-disclosure and the other four variables (self-esteem, perceived stigma, affiliation motives, and subjective well-being). After that, stepwise multiple regression analysis was used to examine whether the self-disclosure domains influenced subjective well-being. The variables exhibiting a significant correlation with subjective well-being were regarded as the independent variables, and subjective well-being (feelings of well-being or feelings of ill-being) was regarded as a dependent variable. All statistical analyses were performed using IBM SPSS Statistics 21.0 (IBM Corporation, Chicago, IL, 2012), and the significance level was set at 0.05. We controlled for multiple comparisons using a false discovery rate (FDR) correction at a threshold of 0.05, following Benjamini-Hochberg [26].

### Ethical considerations

This study was approved by the Sapporo Medical University Ethical Review Board (approval number 25-2-42). The research partnership facilities and each participant provided written and verbal informed consent for all procedures. Their anonymity has been consistently preserved. Overall, this study was conducted according to the Declaration of Helsinki.

## Results

### Participants

Table 1 shows the demographic and clinical characteristics of the participants. The participants were 62 people with SSD (32 men and 30 women, aged 20–65 years old). Their mean age was 44.8 (SD = 10.5); the mean duration of illness was 13.0 years (SD = 2.3); and the mean level of education was 13.0 years (SD = 2.3). Sixty-two were diagnosed with SSD (60 with schizophrenia, 2 with schizoaffective disorder). There was no significant gender difference for age, duration of illness, education, diagnosis, or resident status. All participants were ethnically Japanese.

**Table 1:** Demographics and clinical characteristics of participants.

		Overall (n = 62)	Gender difference		
			Men (n = 32)	Women (n = 30)	P-value^a^
**Age (Mean ± SD)**		44.8 ± 10.5	44.6 ± 10.5	44.9 ± 10.7	0.90
**Duration of illness in years (Mean ± SD)**		18.8 ± 9.4	18.6 ± 9.6	18.9 ± 9.3	0.88
**Education in years (Mean ± SD)**		13.0 ± 2.3	13.0 ± 2.2	13.0 ± 2.4	1.00
**Diagnosis**	**Schizophrenia Schizoaffective disorder**	60 2	31 1	29 1	0.96
**Resident status**	**Single**	24	13	11	0.77
	**Living with family**	27	13	14	
	**Group home**	10	5	5	
	**Others**	1	1	0	

^a^ P-values were derived from an independent t-test for continuous variables and a chi-square test for categorical variables.

### Descriptive statistics, internal consistency, and gender differences

Table 2 shows the characteristics of self-disclosure to first acquaintances and the related gender differences. The sum of each domain was divided by the number of its items, and the results were as follows, in descending order: “living conditions” (2.82), “own strengths” (2.54), “mental illness and psychiatric disability” (2.44), and “experiences of distress” (2.44). The five items with the highest self-disclosure amounts were, again in descending order, “work experience,” “leisure time,” “daily occurrences,” “family relationships,” and “habits,” all included in the domain of “living conditions.” Conversely, the five items with the lowest self-disclosure amounts were, in ascending order, “psychiatric experience,” “own role in society,” “methods of coping with mental illness and psychiatric disability,” “medications for the treatment of mental illness,” and “traumatic experiences.” The all-items score and domain scores in SDMI showed no statistically significant gender differences. Moreover, no individual item had a score showing a statistically significant gender difference after FDR correction. The Cronbach’s alpha coefficient of SDMI (total score and all domains) was within the acceptable range, between 0.894 and 0.969.

**Table 2:** The characteristics of self-disclosure and its gender difference.

	Overall (n = 62)					Men (n = 32)		Women (n = 30)		P-value
	Mean	SD	Median	IR	α	Median	IR	Median	IR	
**SDMI, all items**	**2.56**	**0.95**	**2.57**	**(1.98–3.05)**	0.969	**2.24**	**(1.84–3.03)**	**2.72**	**(2.17–3.17)**	**0.231**
**1. Living Conditions**	**2.82**	**1.04**	**3.00**	**(2.00–3.54)**	0.920	**2.84**	**(1.87–3.33)**	**3.00**	**(2.46–3.83)**	**0.293**
- Income and spending habits	2.35	1.12	2.00	(1.00–3.00)		2.00	(1.00–3.00)	2.50	(1.75–3.25)	0.183
- Habits	2.81 ^*a*^	1.24	3.00	(2.00–4.00)		2.50	(2.00–4.00)	3.00	(2.00–4.00)	0.643
- Family relationships	2.84 ^*a*^	1.30	3.00	(2.00–4.00)		2.00	(1.25–4.00)	3.00	(2.00–4.00)	0.327
- Leisure time	2.97 ^*a*^	1.20	3.00	(2.00–4.00)		3.00	(2.00–4.00)	3.00	(2.00–4.00)	0.143
- Daily occurrences	2.97 ^*a*^	1.23	3.00	(2.00–4.00)		3.00	(2.00–3.75)	3.00	(3.00–4.00)	0.079
- Work experience	3.00 ^*a*^	1.32	3.00	(2.00–4.00)		3.00	(2.00–4.00)	3.00	(2.00–4.00)	0.717
**2. Own strengths**	**2.54**	**0.99**	**2.60**	**(1.95–3.05)**	0.894	**2.30**	**(1.80–2.95)**	**2.80**	**(2.00–3.20)**	**0.109**
- Own role in society	2.32 ^*b*^	1.02	2.00	(2.00–3.00)		2.00	(1.25–3.00)	2.00	(2.00–3.00)	0.369
- Own growth	2.45	1.24	2.00	(1.00–3.00)		2.00	(1.00–3.00)	3.00	(2.00–4.00)	0.025
- Motivation	2.60	1.25	2.00	(2.00–3.25)		2.00	(1.25–3.00)	2.50	(2.00–4.00)	0.566
- Own abilities and skills	2.65	1.16	3.00	(2.00–3.25)		2.00	(2.00–3.75)	3.00	(2.00–3.25)	0.755
- Dreams and goals for the future	2.66	1.23	2.00	(2.00–4.00)		2.00	(2.00–3.75)	3.00	(2.00–4.00)	0.126
**3. Mental illness and psychiatric disability**	**2.44**	**1.17**	**2.17**	**(1.33–3.33)**	0.949	**2.00**	**(1.21–3.67)**	**2.50**	**(1.54–3.33)**	**0.507**
- Methods of coping with mental illness and psychiatric disability	2.32 ^*b*^	1.24	2.00	(1.00–3.00)		2.00	(1.00–3.00)	2.00	(1.00–3.00)	0.540
- Medications for the treatment of mental illness	2.32 ^*b*^	1.36	2.00	(1.00–3.00)		2.00	(1.00–3.75)	2.00	(1.00–3.00)	0.965
- Effectiveness of medications	2.37	1.38	2.00	(1.00–3.25)		2.00	(1.00–4.00)	2.00	(1.00–3.00)	0.798
- Mental illness	2.48	1.29	2.00	(1.00–3.25)		2.00	(1.00–3.75)	2.00	(1.75–3.25)	0.621
- Psychiatric symptoms and disorders	2.52	1.29	2.00	(1.00–4.00)		2.00	(1.00–4.00)	2.50	(2.00–4.00)	0.458
- Experience with psychiatric services	2.61	1.31	2.00	(2.00–4.00)		2.00	(2.00–3.75)	3.00	(1.75–4.00)	0.323
**4. Experience of distress**	**2.44**	**1.02**	**2.33**	**(1.67–3.04)**	0.917	**2.17**	**(1.50–2.83)**	**2.59**	**(1.79–3.21)**	**0.156**
- Psychiatric experiences	2.27 ^*b*^	1.26	2.00	(1.00–3.00)		2.00	(1.00–2.75)	2.00	(1.00–3.00)	0.628
- Traumatic experiences	2.32 ^*b*^	1.28	2.00	(1.00–3.00)		2.00	(1.00–3.00)	2.00	(2.00–4.00)	0.034
- Experiences of devaluation and discrimination	2.44	1.25	2.00	(1.00–3.00)		2.00	(1.00–3.00)	2.00	(2.00–4.00)	0.195
- Problems involving interpersonal relationships	2.55	1.18	2.00	(2.00–3.00)		2.00	(1.00–3.00)	2.50	(2.00–4.00)	0.228
- Problems involving living environment	2.63	1.16	2.50	(2.00–3.25)		2.00	(2.00–3.00)	3.00	(2.00–4.00)	0.546
- Health problems	2.69	1.14	3.00	(2.00–4.00)		2.00	(2.00–4.00)	3.00	(2.00–3.25)	0.561

Note. SDMI: Self-Disclosure scale for people with Mental Illness, SD: standard deviation, IR: interquartile range,α: Cronbach’s alpha coefficient

^*a*^ Five items with the highest self-disclosure amounts

^*b*^ Five items with the lowest self-disclosure amounts

Table 3 shows the descriptive statistics and internal consistency for the Rosenberg self-esteem scale, Link devaluation-discrimination scale, questionnaires on affiliation motives, and SUBI. In examining gender difference for each scale, feelings of well-being were demonstrated at a significantly lower scale for men than women. The other variables showed no statistically significant gender differences. The Cronbach’s alpha coefficient of each scale was within the acceptable range, between 0.810 and 0.905.

**Table 3:** Description and internal consistency of other variables.

	Overall (n = 62)					Men (n = 32)		Women (n = 30)		P-value
	Mean	SD	Median	IR	α	Median	IR	Median	IR	
**Rosenberg self-esteem scale**	28.66	7.56	29.50	24.75–33.00	0.836	30.00	24.00–33.00	29.00	25.75–33.00	0.724
**Link devaluation-discrimination scale**	33.23	5.87	33.50	29.00–37.00	0.810	34.00	28.25–38.00	33.00	29.00–35.25	0.534
**Questionnaires on affiliation motives—Affiliation tendency**	32.23	7.16	32.00	28.00–37.25	0.880	32.00	27.00–37.00	32.00	29.00–38.50	0.611
** -Sensitivity to rejection**	31.90	7.67	33.00	27.00–37.00	0.890	31.50	27.25–37.75	33.50	26.50–36.00	0.905
**SUBI—Feelings of well-being**	34.05	7.68	34.50	27.75–40.25	0.905	30.00	25.25–36.75	37.00	31.00–41.75	0.003*
**- Feelings of ill-being**	46.35	6.71	46.00	43.00–51.00	0.857	44.50	40.00–50.75	46.50	43.00–50.25	0.405

Note: SUBI: Subjective well-being inventory, SD: standard deviation, IR: interquartile range,α: Cronbach’s alpha coefficient

* Significant after false discovery rate correction

### Relationship between self-disclosure and the four variables

Table 4 shows the correlation coefficients between self-disclosure, well-being, and four variables: self-esteem, perceived stigma, affiliation motives, and subjective well-being. For all participants, the total score of self-disclosure was significantly correlated with feelings of well-being (ρ = 0.360, p < 0.01) after FDR correction. Analysis of self-disclosure domains showed that self-disclosure about “living conditions” was significantly correlated with feelings of well-being (ρ = 0.428, p < 0.01) and that self-disclosure about “own strengths” was significantly correlated with self-esteem (ρ = 0.394, p < 0.01) and feelings of well-being (ρ = 0.462, p < 0.001) after FDR correction. However, self-disclosure about “mental illness and psychiatric disability” and “experiences of distress” were not significantly correlated with any of the variables after FDR correction.

**Table 4:** The correlation coefficients between self-disclosure and other variables.

		Rosenberg self-esteem scale		Link devaluation-discrimination scale		Questionnaires on affiliation motives				SUBI			
						Affiliation tendency		Sensitivity to rejection		Feelings of well-being		Feelings of ill-being	
		ρ	P-value	ρ	P-value	ρ	P-value	ρ	P-value	ρ	P-value	ρ	P-value
**All**	**SDMI all items**	0.317	0.012	-0.222	0.082	0.276	0.030	0.233	0.068	0.360	0.004*	0.202	0.115
	**- Living Conditions**	0.315	0.013	-0.178	0.166	0.252	0.048	0.243	0.057	0.428	0.001*	0.183	0.155
	**- Own Strengths**	0.394	0.002*	-0.229	0.074	0.155	0.230	0.211	0.100	0.462	<0.001*	0.281	0.027
	**- Mental Illness and Psychiatric Disability**	0.194	0.131	-0.173	0.179	0.239	0.061	0.234	0.068	0.188	0.143	0.136	0.291
	**- Experiences of Distress**	0.324	0.010	-0.216	0.092	0.210	0.101	0.220	0.085	0.286	0.024	0.195	0.129
**Men**	**SDMI all items**	0.319	0.075	-0.245	0.177	0.403	0.022	0.179	0.327	0.227	0.221	0.495	0.004*
	**- Living Conditions**	0.350	0.050	-0.219	0.228	0.382	0.031	0.273	0.131	0.322	0.072	0.498	0.004*
	**- Own Strengths**	0.442	0.011	-0.333	0.063	0.200	0.273	0.200	0.273	0.276	0.126	0.546	0.001*
	**- Mental Illness and Psychiatric Disability**	0.214	0.240	-0.134	0.463	0.389	0.028	0.169	0.354	0.122	0.507	0.420	0.017
	**- Experiences of Distress**	0.283	0.117	-0.204	0.262	0.354	0.047	0.115	0.529	0.093	0.613	0.484	0.005*
**Women**	**SDMI all items**	0.296	0.112	-0.133	0.483	0.053	0.782	0.234	0.213	0.466	0.010	-0.202	0.286
	**- Living Conditions**	0.267	0.153	-0.082	0.665	0.118	0.534	0.163	0.391	0.553	0.002*	-0.262	0.163
	**- Own Strengths**	0.280	0.134	-0.030	0.875	0.125	0.510	0.212	0.261	0.630	<0.001*	-0.093	0.624
	**- Mental Illness and Psychiatric Disability**	0.151	0.427	-0.146	0.442	0.031	0.870	0.310	0.095	0.264	0.158	-0.265	0.157
	**- Experiences of Distress**	0.330	0.075	-0.160	0.400	0.033	0.861	0.332	0.073	0.434	0.017	-0.218	0.248

Note: SDMI: Self-disclosure scale for people with mental illness; SUBI: Subjective well-being inventory

* Significant after false discovery rate correction

Next, the correlation coefficients were analyzed by gender. The data for men indicated a correlation between the total score of self-disclosure and feelings of ill-being (ρ = 0.495, p < 0.01) after FDR correction. The self-disclosure domains associated with feelings of ill-being were self-disclosure of “living conditions” (ρ = 0.498, p < 0.01), “own strengths” (ρ = 0.546, p < 0.01), and “experiences of distress” (ρ = 0.484, p < 0.01) after FDR correction. However, self-disclosure about “mental illness and psychiatric disability” had no correlation with feelings of ill-being after FDR correction. By contrast, the data for women indicated no correlation between the total score of self-disclosure and each variable after FDR correction. However, the self-disclosure domains associated with feelings of well-being were self-disclosure about “living conditions” (ρ = 0.553, p < 0.01) and “own strengths” (ρ = 0.630, p < 0.001), but self-disclosure about “mental illness and psychiatric disability” and “experiences of distress” had no correlation with feelings of well-being after FDR correction.

### Self-disclosure domains relate to subjective well-being in a stepwise multiple regression analysis

Table 5 shows the domains of self-disclosure, self-esteem, perceived stigma, and affiliation motives, which were significantly associated with the SUBI subscales in stepwise multiple regression analysis. Since the correlations between the independent variables were not strong (|ρ| < 0.90), stepwise multiple regression analyses were performed.

**Table 5:** Factors associated with variables of subjective well-being in a stepwise multiple regression analysis.

	Dependent variable	Independent variable	β	P-value
**Men**	Feelings of Well-being	Self-Esteem	0.450	0.007*
		Perceived Stigma Adjusted R^2^ = 0.378	-0.320	0.047 <0.001*
	Feeling of Ill-being	Self-Disclosure (Living Conditions)	0.308	0.030*
		Self-Disclosure (Own Strengths)	-0.230	0.396
		Self-Disclosure (Mental Illness and Psychiatric Disability)	0.076	0.659
		Self-Disclosure (Experiences of Distress)	0.148	0.506
		Self-Esteem	0.392	0.011*
		Perceived Stigma Adjusted R^2^ = 0.523	-0.306	0.032* <0.001*
**Women**	Feelings of Well-being	Self-Disclosure (Living Conditions)	0.142	0.512
		Self-Disclosure (Own Strengths)	0.420	0.001*
		Self-Disclosure (Experiences of Distress)	-0.236	0.152
		Self-Esteem	0.548	<0.001*
		Affiliation motives (Sensitivity to rejection) Adjusted R^2^ = 0.701	0.400	0.001* <0.001*
	Feeling of Ill-being	Self-Esteem Adjusted R^2^ = 0.151	0.424	0.019* 0.019*

*Significant after false discovery rate correction

For men, the factor retained in the model for feelings of well-being was self-esteem (β = 0.450, p < 0.01). The fitness of the model was low (Adjusted R^2^ = 0.378, p < 0.001). The factors retained in the model for feelings of ill-being were self-disclosure about “living conditions” (β = 0.308, p < 0.05), self-esteem (β = 0.392, p < 0.05) and perceived stigma (β = -0.306, p < 0.05). The fitness of the model was good (Adjusted R^2^ = 0.523, p < 0.001).

For women, the factors retained in the model for feelings of well-being were self-disclosure about “own strengths” (β = 0.420, p < 0.01), self-esteem (β = 0.548, p < 0.01), and the sensitivity to rejection of affiliation motives (β = 0.400, p < 0.01). The fitness of the model was good (Adjusted R^2^ = 0.701, p < 0.001). The factor retained in the model for feelings of well-being was self-esteem (β = 0.424, p < 0.05). The fitness of the model was low (Adjusted R^2^ = 0.151, p < 0.05).

## Discussion

This study aimed to examine the characteristic features of and gender differences in self-disclosure to first acquaintances and to clarify the relationship between self-disclosure and subjective well-being. The present results indicate that, with first acquaintances, self-disclosure concerning living conditions is the highest domains indicating “partial disclosure,” whereas that related to experience of illness and distress is the lowest indicating domains indicating almost “little disclosure”. No gender differences were found for the self-disclosure domains. From the multiple regression analyses according to identified characteristics for each gender, men’s feelings of ill-being were related to self-disclosure about “living conditions,” self-esteem, and perceived stigma; by contrast, women’s feelings of well-being were related to self-disclosure about “own strengths,” self-esteem, and sensitivity to rejection.

This is the first study to investigate self-disclosure to first acquaintances. The first key point of this study is the amount of self-disclosure. Based on the results, we formulated the following hypothesis: in each of the four domains, people with SSD living in the community tend to self-disclose to first acquaintances less frequently than that to close people, in accordance with our previous findings [11]. This hypothesis supports a social penetration model [14], which claims that the breadth and depth of self-disclosure are more likely to increase with the building of personal relationships.

The second key point of this study is the relationships between self-disclosure domains and subjective well-being. Self-disclosure to first acquaintances regarding “living conditions” and “own strengths” are positively correlated with subjective well-being, but self-disclosure regarding “mental illness and disability” and “experiences of distress” are not. This differs from our previous finding that subjective well-being is related to all domains of self-disclosure to close people [13]. This study suggests only that the act of disclosing these matters to first acquaintances was more likely to be reported by those with higher ratings of well-being. It is critical that people with SSD are accepted when disclosing their experiences as human beings, such as their “living conditions” and “own strengths,” when encountering anyone in community life. The psychological recovery model [27] states that hope and self-determination lead to a meaningful life and a positive sense of self, regardless of the presence of mental illness. The strengths model [28] focuses on people’s strengths rather than their deficits. It demonstrates the importance of their passions, skills, interests, relationships, and environments. The present results are expected to support the recovery and strengths models. However, they do not necessarily suggest that people with SSD should disclose the self on first meeting a new acquaintance. It is important to disclose aspects of oneself at an appropriate time in the formation of interpersonal relationships.

This study also investigated gender differences as they relate to self-disclosure. For the self-disclosure total score and domain scores, no significant gender differences were found. These results were not in line with those reported by a previous study of undergraduates [9]. It can be presumed that participants would tend to disclose neither about their illness/disability nor about details of their life experiences resulting from mental illness/disability regardless of gender. Internationally, mental health stigma is a central issue for people with mental illness [3,11,12], and it is a likely influence on low subjective well-being and the avoidance of interpersonal relations. There is a weak negative association between perceived stigma and self-disclosure to close persons [13]. In addition, deficits in metacognitive processes [29,30], such as navigating interpersonal relationships and first-person experiences in the moment, and overlooking own positive aspects and motives, might lead to lesser degrees of self-disclosure. However, contrary to expectations, the present study indicated that self-disclosure to first acquaintances was not related to perceived stigma, but rather, to self-esteem and affiliation tendency. Therefore, in the early stages of interpersonal relationships, self-disclosure, rather than the level of perceived stigma, could be a major contributor to a feeling of worthiness to “be human” and a desire to form relationships regardless of illness or disability.

Multiple regression analyses by gender revealed the relationship between self-disclosure and subjective well-being. Among men, self-disclosure about “living conditions” showed a significant correlation with feelings of ill-being, as did self-esteem and perceived stigma. The present results thus suggest that self-disclosing examples of daily events, habits, and leisure time pursuits could be related to relieving perceived ill health and negative affect. Men more strongly predicted negative ramifications of disclosure than did women, such as feeling vulnerable, feeling uncomfortable, having a weakness exposed, and being rejected by the person to whom they reveal information [31]. However, men reported being more willing than women to disclose to strangers and acquaintances [9]. Men may be motivated to form interpersonal relationships through self-disclosure as a result of the elimination of negative ramifications.

For women, self-disclosure about “own strengths” showed a significant correlation with feelings of well-being, as did self-esteem and affiliation tendency. The present results thus suggest that self-disclosure of examples of motivation, self-growth, and personal goals could be related to subjective well-being, for example, through expectation-achievement congruence and transcendence. Women more strongly predicted positive ramifications than did men, such as the clarification of information about the self and increases in intimacy, trust levels, satisfaction, and feelings of acceptance by a target person [31]. Relatedly, women with schizophrenia spectrum disorders have previously reported higher positive mental health, such as “emotional support” and “personal growth and autonomy,” compared to men [32]. In this respect, it is possible that women have a more positive attitude to self-disclosure than men. Positive attitude helps to enhance the relationship between self-disclosure about one’s own growth and subjective well-being. As above, these correlations are in the same direction for both genders, though the qualities of well-being related to self-disclosure differed by gender. It may be effective for the therapist to understand gender differences in each aspect of self-disclosure.

The present study has several limitations. First, the small sample size and exclusive focus on Japanese culture may limit the generalizability of results. Second, this study cannot determine causality due to its cross-sectional design. The multiple regression analysis should, therefore, be treated as a pilot study on which future research can build through a longitudinal design, using mediation analysis or path analysis, including self-disclosure and subjective well-being, to advance the interpretation of results. Third, the self-disclosure scale was measured by participants recalling meetings with first acquaintances. Therefore, it might not accurately measure actual self-disclosure. In addition, multicenter studies will be necessary to investigate both subjective and objective self-disclosure. Fourth, the influence of self-disclosure on age trends was not taken into consideration. A previous study clarifies the age trends in self-disclosure amount with regard to the target person [33]. In the present study, participants were aged from 20 to 65 years, but it is necessary to investigate the influence of age on self-disclosure.

In conclusion, our findings suggest that self-disclosure to first acquaintances is related to subjective well-being in people with SSD. The self-disclosure domains most strongly related to subjective well-being differed by gender: “living conditions” for men and “own strengths” for women. On the other hand, this study suggests that a lack of disclosure is related to negative effects on quality of life. This study’s impact is its focus on the importance of self-disclosure in the context of psychiatric rehabilitation, encouraging therapists to support people with SSD to practice self-disclosure to first acquaintances as a method of recovery, which can be implemented using psychosocial intervention. Therapists can establish a therapeutic group, such as through group therapy or social skills training, in which individuals can disclose their living conditions and their own strengths to first acquaintances in community-based rehabilitation. At the same time, it is necessary to respect their self-disclosure and provide support that emphasizes individuality. We believe that self-disclosure is important in SSD recovery from the client’s point of view, and clients should not be compelled into disclosure at the initiative of the therapist. Timing and styles for promoting self-disclosure should vary between individuals.

## Supporting information

S1 Appendix English version of the self-disclosure scale for people with Mental Illness (SDMI). (DOCX)
