# Correction: Relationship between self-disclosure to first acquaintances and subjective well-being in people with schizophrenia spectrum disorders living in the community

**DOI:** 10.1371/journal.pone.0225680

**Published:** 2019-11-19

**Authors:** The PLOS ONE Staff

There are errors in Tables 1 and 5. The publisher apologizes for these errors. Please see the correct Tables 1 and 5 here.

**Table 1:** Demographics and clinical characteristics of participants.

		Overall (n = 62)	Gender difference		P-value^a^
			Men (n = 32)	Women (n = 30)	
**Age (Mean ± SD)**		44.8 ± 10.5	44.6 ± 10.5	44.9 ± 10.7	0.90
**Duration of illness in years (Mean ± SD)**		18.8 ± 9.4	18.6 ± 9.6	18.9 ± 9.3	0.88
**Education in years (Mean ± SD)**		13.0 ± 2.3	13.0 ± 2.2	13.0 ± 2.4	1.00
**Diagnosis**	**Schizophrenia**	60	31	29	0.96
	**Schizoaffective disorder**	2	1	1	
**Resident status**	**Single**	24	13	11	0.77
	**Living with family**	27	13	14	
	**Group home**	10	5	5	
	**Others**	1	1	0	

^a^ P-values were derived from an independent t-test for continuous variables and a chi-square test for categorical variables.

**Table 5:** Factors associated with variables of subjective well-being in a stepwise multiple regression analysis.

	Dependent variable	Independent variable	β	P-value
**Men**	Feelings of Well-being	Self-Esteem	0.450	0.007*
		Perceived Stigma	-0.320	0.047
		Adjusted R^2^ = 0.378		<0.001*
	Feeling of Ill-being	Self-Disclosure (Living Conditions)	0.308	0.030*
		Self-Disclosure (Own Strengths)	-0.230	0.396
		Self-Disclosure (Mental Illness and Psychiatric Disability)	0.076	0.659
		Self-Disclosure (Experiences of Distress)	0.148	0.506
		Self-Esteem	0.392	0.011*
		Perceived Stigma	-0.306	0.032*
		Adjusted R^2^ = 0.523		<0.001*
**Women**	Feelings of Well-being	Self-Disclosure (Living Conditions)	0.142	0.512
		Self-Disclosure (Own Strengths)	0.420	0.001*
		Self-Disclosure (Experiences of Distress)	-0.236	0.152
		Self-Esteem	0.548	<0.001*
		Affiliation motives (Sensitivity to rejection)	0.400	0.001*
		Adjusted R^2^ = 0.701		<0.001*
	Feeling of Ill-being	Self-Esteem	0.424	0.019*
		Adjusted R^2^ = 0.151		0.019*

*Significant after false discovery rate correction
