# Supplementary material for: Relationship between self-disclosure to first acquaintances and subjective well-being in people with schizophrenia spectrum disorders living in the community
Source: PLoS One. 2019 Oct 16;14(10):e0223819. doi: 10.1371/journal.pone.0223819 (PMC6795466; doi:10.1371/journal.pone.0223819)
Supplement: S1 Appendix — (DOCX) [file pone.0223819.s001.docx]

**S1 Appendix. English version of the Self-Disclosure scale for people with Mental Illness (SDMI)**

This questionnaire is used to ascertain the amounts of self-disclosure of people with mental disorders. Think of “first acquaintances” with whom you will continue to have a relationship in the future. Consider the amounts of self-disclosure (how much you do/do not discuss a topic) with “first acquaintances.” Please circle the applicable number for each topic　(see Table A). If you find something that does not apply to your life, leave it blank. (It does not affect the results, as the average score is calculated).

**Table A. English version of the SDMI**

|  | No disclosure | Little disclosure | Partial disclosure | Moderate disclosure | Full disclosure |
| --- | --- | --- | --- | --- | --- |
| **1. Living Conditions** |  |  |  |  |  |
| - Leisure time | 1 | 2 | 3 | 4 | 5 |
| - Habits | 1 | 2 | 3 | 4 | 5 |
| - Daily occurrences | 1 | 2 | 3 | 4 | 5 |
| - Family relationships | 1 | 2 | 3 | 4 | 5 |
| - Work experience | 1 | 2 | 3 | 4 | 5 |
| - Income and spending habits | 1 | 2 | 3 | 4 | 5 |
| **2. Own strengths** |  |  |  |  |  |
| - Motivation | 1 | 2 | 3 | 4 | 5 |
| - Own growth | 1 | 2 | 3 | 4 | 5 |
| - Dreams and goals for the future | 1 | 2 | 3 | 4 | 5 |
| - Own abilities and skills | 1 | 2 | 3 | 4 | 5 |
| - Own role in society | 1 | 2 | 3 | 4 | 5 |
| **3. Mental illness and psychiatric disability** |  |  |  |  |  |
| - Mental illness | 1 | 2 | 3 | 4 | 5 |
| - Psychiatric symptoms and disorders | 1 | 2 | 3 | 4 | 5 |
| - Methods of coping with mental illness and psychiatric disability | 1 | 2 | 3 | 4 | 5 |
| - Medications for the treatment of mental illness | 1 | 2 | 3 | 4 | 5 |
| - Effectiveness of medications | 1 | 2 | 3 | 4 | 5 |
| - Experience with psychiatric services | 1 | 2 | 3 | 4 | 5 |
| **4. Experience of distress** |  |  |  |  |  |
| - Problems involving interpersonal relationships | 1 | 2 | 3 | 4 | 5 |
| - Problems involving living environment | 1 | 2 | 3 | 4 | 5 |
| - Health problems | 1 | 2 | 3 | 4 | 5 |
| - Traumatic experiences | 1 | 2 | 3 | 4 | 5 |
| - Psychiatric experiences | 1 | 2 | 3 | 4 | 5 |
| - Experiences of devaluation and discrimination | 1 | 2 | 3 | 4 | 5 |
